# Computational Fluid Dynamics Could Enable Individualized Surgical Treatment of Nasal Obstruction (A Preliminary Study)

**DOI:** 10.3390/diagnostics12112642

**Published:** 2022-10-31

**Authors:** Marek Plášek, Michaela Masárová, Marián Bojko, Pavel Komínek, Petr Matoušek, Martin Formánek

**Affiliations:** 1 Department of Otorhinolaryngology and Head and Neck Surgery, University Hospital Ostrava, 17. Listopadu 1790, 70852 Ostrava, Czech Republic; 2 Faculty of Medicine, University of Ostrava, 70300 Ostrava, Czech Republic; 3 Faculty of Mechanical Engineering, Technical University of Ostrava, 17. Listopadu 2172/15, 70800 Ostrava, Czech Republic

**Keywords:** computational fluid dynamics, nasal airflow, 3D model, nasal surgery, planning

## Abstract

Passage of nasal airflow during breathing is crucial in achieving accurate diagnosis and optimal therapy for patients with nasal disorders. Computational fluid dynamics (CFD) is the dominant method for simulating and studying airflow. The present study aimed to create a CFD nasal airflow model to determine the major routes of airflow through the nasal cavity and thus help with individualization of surgical treatment of nasal disorders. The three-dimensional nasal cavity model was based on computed tomography scans of the nasal cavity of an adult patient without nasal breathing problems. The model showed the main routes of airflow in the inferior meatus and inferior part of the common meatus, but also surprisingly in the middle meatus and in the middle part of the common nasal meatus. It indicates that the lower meatus and the lower part of the common meatus should not be the only consideration in case of surgery for nasal obstruction in our patient. CFD surgical planning could enable individualized precise surgical treatment of nasal disorders. It could be beneficial mainly in challenging cases such as patients with persistent nasal obstruction after surgery, patients with empty nose syndrome, and patients with a significant discrepancy between the clinical findings and subjective complaints.

## 1. Introduction

From an aerodynamic point of view, the nasal cavity is a complex structure with a series of flexures and narrow spaces that air flows around and through. Moreover, as air flows through the nasal cavity, it is humidified, heated, and purified. These essential functions require correct contact between the air flowing in and the nasal mucosa [1,2].

Nasal pathologies (e.g., septal deviations/perforations, mucosal hypertrophy) can disturb the nasal airflow. They are often inconsistent with the patient’s subjective complaints. Some people with significant anatomical variations do not feel any nasal obstruction. Conversely, patients that experience anatomical changes that are objectively considered small might complain of significant nasal obstruction [3,4]. The situation is similar in patients with empty nose syndrome. Thus, it is likely that anatomical features are not the only factors involved in the sensation of nasal disorders. In these patients, nasal disorders might be caused by changes in the nasal airflow, or alternatively, changes in the nasal cycle. These aerodynamic changes are difficult to detect with conventional methods, such as rhinomanometry, acoustic rhinometry, or peak nasal inspiratory flow, because the complex anatomy of the nasal cavity does not allow a detailed study of nasal aerodynamics. Therefore, the study of nasal airflow has focused on the use of model simulations [5,6].

Many different methods have been developed previously to determine airflow and other variables in the nasal cavity. Currently, computational fluid dynamics (CFD) is the dominant method for simulating and studying nasal airflow patterns. In 1995, a 3D computer-generated model was described that simulated airflow in the nose, based on computed tomography (CT) scan results. Since then, computational fluid dynamics modeling has been used to study the localization of airflow and the physical parameters of flow (velocity, flow pattern and distribution, etc.), in both physiological and pathological conditions [6,7]. Knowledge of how air flows through the nasal cavities during breathing is crucial in achieving accurate diagnostics and optimal therapy for patients with nasal or paranasal disorders.

The present study aimed to create a computational fluid dynamics model of nasal airflow to determine the major routes of airflow through the nasal cavity, which could possibly enable surgical planning and individualized surgical treatment focused on particular parts of the nasal cavity during rhinosurgery.

## 2. Methods

The study was conducted in accordance with the Declaration of Helsinki, and approved by the Ethics Committee of University Hospital Ostrava (2017). 

### 2.1. Virtual Model of the Nasal Cavity

**Figure 1:**
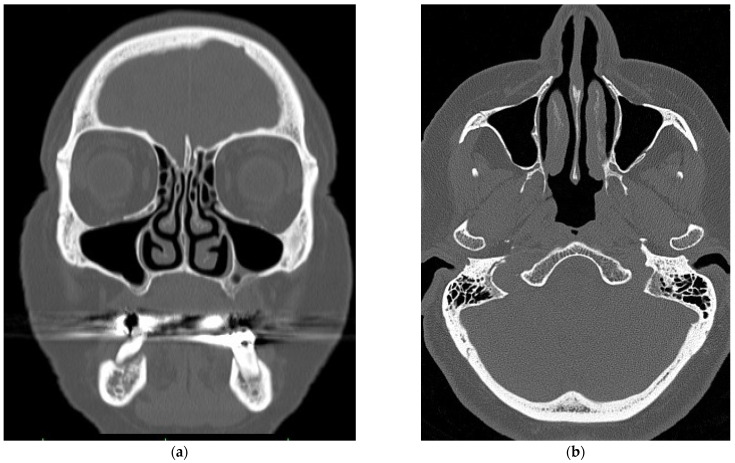
CT images of the nasal cavity—(**a**) frontal section (**b**) transverse section.

**Figure 2:**
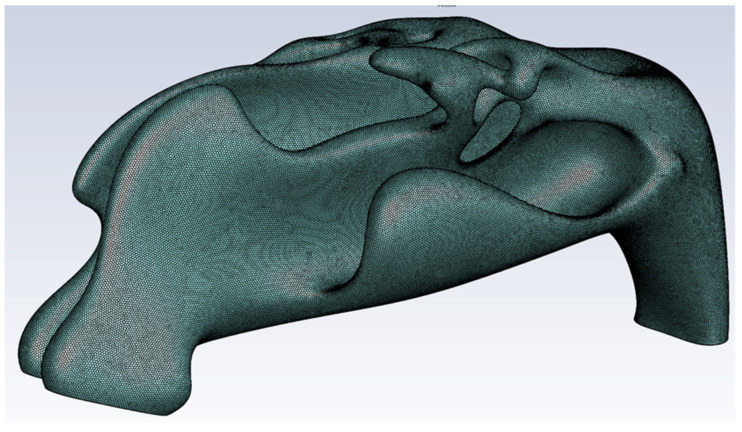
3D model of the nasal cavity.

**Figure 3:**
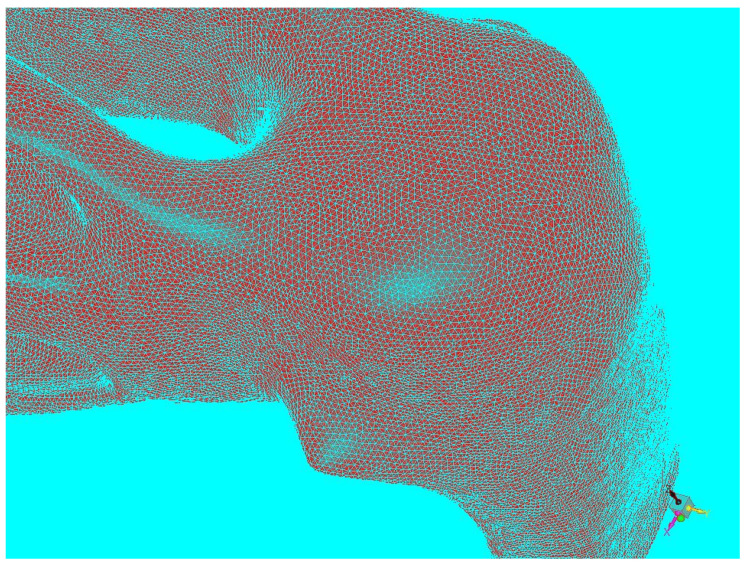
Computational network of a 3D model.

We collected computed tomography scans (CT Siemens, Siemens Healthineers AG, Erlangen, Germany) of the nasal cavity (cut in 1 mm sections) performed on a female patient (age 64 years, height 173 cm, weight 75 kg) at the Department of Otorhinolaryngology and Head and Neck Surgery of the University Hospital Ostrava, Czech Republic. The patient had no problem with nasal breathing, and no pathologies, like nasal septal deviations/perforations, were detected during clinical examination or on the computed tomography scans (Figure 1a,b). Therefore, the nasal cavity was considered “physiologically normal” (the CT scans had been done due to transnasal pituitary surgery). We created a virtual 3D model of the nasal cavity based on the data from the computed tomography scans in cooperation with the VSB—Technical University of Ostrava, Czech Republic (Figure 2). We imported the model into the ANSYS Design Modeler environment (ANSYS, Southpointe, PA, USA) and created a computational network for subsequent computational fluid dynamics simulations (Figure 3).

### 2.2. Nasal Airflow Simulation

We simulated airflow during inspiration with constant physical attributes. The air was isothermic, incompressible, and we did not include the effect of gravity. The walls of the model were firm, without considering movement or deformations; in other words, we did not consider mucosal changes. The pattern of airflow was laminar, and the pressure gradient was 120 Pa between the entrance (nasal entry) and the exit (nasal choana).

### 2.3. Locations of Measurements

**Figure 4:**
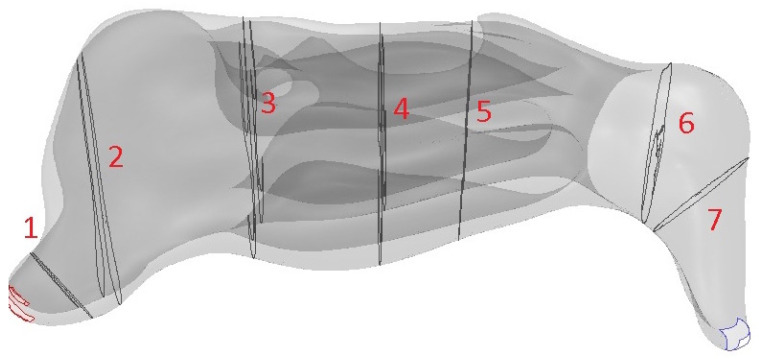
Model simulations of frontal sections 1–7 in the nasal cavity.

To assess the aerodynamics in individual parts of the nasal cavity and to determine possible changes during passage through the nasal cavity, we defined the following 7 regions for evaluation (sections of the frontal plane of CT images; Figure 4):

1. The nasal entrance (at 5 mm behind the entrance to the nasal cavity).
2. The nasal valve (the plane that passed through the anterior edge of the lateral nasal cartilage).
3. The front part of the nasal turbinates (at 5 mm behind the start of the lower turbinate),
4. The middle part of the nasal turbinates (at 25 mm behind the start of the lower turbinate).
5. The posterior part of the nasal turbinates (at 35 mm behind the start of the lower turbinate).
6. The choana (the plane that passed through the choana, at 88 mm behind the nasal cavity entrance).
7. The nasopharynx (the plane in the nasopharyngeal area, at 97 mm behind the nasal cavity entrance).

We used the traditional anatomical definitions of the different parts of the nasal meatus for the evaluations. The common meatus was between the septum and nasal turbinates; the lower meatus was the space below the lower turbinate; the middle meatus was the space under the middle turbinate. The upper meatus was the space between the superior and the middle turbinate.

## 3. Results

### 3.1. The Main Routes of Nasal Airflow

**Figure 5:**
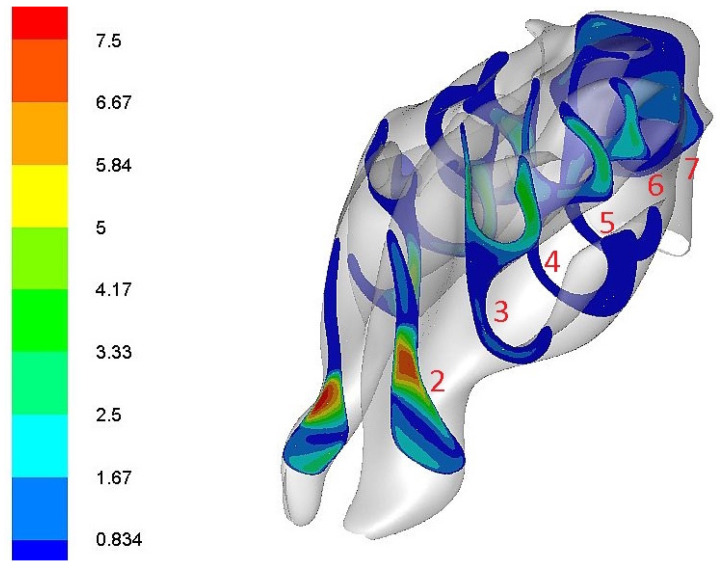
Evaluation of airflow (cm^3^/s) in frontal sections 2–7 in the nasal cavity. Distribution of airflow is color-coded: blue—the lowest; red—the highest.

In the left nasal cavity, the dominant flow (cm^3^/s) was directed to the upper portion of the common meatus in the entrance and in the center regions of the model. In the right nasal cavity, the main airflow was directed to the lower and middle portions of the common meatus. Subsequently, in the posterior area of the model, the airflow was concentrated more toward the middle meatus and the middle portions of the common nasal meatus in both nasal cavities (Figure 5).

### 3.2. The Greatest Resistance to Airflow

The magnitude of the resistance to airflow was equal to the drop in pressure as the air flowed through the nasal passages. We evaluated the percentage difference in pressures between the individual sections. Among the evaluated differential pressures, in both nasal cavities we measured the highest resistance in the area of the nasal valve, between planes 1 (nasal entrance), 2 (nasal valve), and 3 (anterior portion of the nasal turbinates). The resistance decreased almost constantly towards the nasopharynx (except the area between the posterior part of the nasal turbinates and the choana). The lowest resistance was detected between the choana and the nasopharynx (Table 1).

### 3.3. Highest Airflow Velocity

The highest airflow velocity was 11.8 m/s, and it occurred in the plane of the nasal entrance. The airflow slowed down as it passed through the nasal cavity, and its velocity in the area of the nasal valve was 8.3 m/s. In the area of the anterior part of the nasal turbinates, the maximum speed was 4 m/s (approximately half). This occurred at different spots in the right and left nasal cavity. In the left nasal cavity, it occurred in the middle meatus. In the right nasal cavity, it occurred in middle part of the common meatus. In the plane of the central part of the nasal turbinates, the airflow decelerated further to 3.7 m/s. In the rear part of the nasal turbinates, the velocity was only about 2 m/s. In the area of the nasopharynx, the velocity was about 1 m/s, and it was evenly distributed over the cross section (Table 2).

## 4. Discussions

The main advantage of computational fluid dynamics is that it allows simulations of airflow in physiological and many pathological conditions, without requiring many different models (e.g., mechanical models) [8,9]. Computational fluid dynamics can be characterized as numerical modelling. Many software programs can be used for modeling. We worked with the ANSYS Fluent program (ANSYS, USA). To interpret the results correctly, it was crucial to define input and output data and to define the locations for taking measurements [10,11].

To diagnose and treat patients with nasal disorders, it is crucial to understand the character of nasal airflow and the routes taken by the majority of the air flowing through the nose during breathing. However, there is no consensus on which part of nasal cavity receives the majority of airflow. According to Simmen et al., the majority of airflow was detected in the middle meatus and in the middle part of the common meatus [12]. In contrast, Mlynski et al. detected the majority of airflow in the lower meatus and in the lower part of the common meatus [13].

In our study, the majority of airflow passed through different parts of the left and right nasal cavities. In the left nasal cavity, in the entrance and middle part, the majority of airflow was detected in the upper part of the common nasal meatus; in the right nasal cavity, in the entrance and middle part, the majority of airflow was detected in the inferior and middle parts of the common meatus. Posteriorly, the main airflow was concentrated in the middle meatus and the middle part of the common nasal meatus in both nasal cavities. The highest resistance was measured in the area of the nasal valve in both nasal cavities. The highest velocity was detected in the plane of the nasal entrance in both nasal cavities.

The different pattern of nasal airflow between left and right cavity was simulated also in Zhao et al. [14]. In two previous studies, Zhao et al. demonstrated that there was more than one physiological pattern of nasal airflow, and also declared great inter-individual variability among healthy adults [14,15]. In addition, our finding that the highest resistance in the nasal cavity was in the area of nasal valve was consistent with previous results reported by Wang et al. [16].

Currently, surgical therapy for nasal obstructions (septoplasty, turbinoplasty) is particularly focused on the inferior meatus and the inferior part of the common nasal meatus. Our results showed that the majority of airflow was detected also in the middle parts of nasal cavities. This finding gave rise to the question of whether the middle meatus should be considered in surgical therapy for nasal obstructions as well. This notion was supported by a previous study from Tan et al., who detected only small proportions of the air stream in the lower meatus and the majority of flow through the middle meatus [17]. Moreover, Lee et al. simulated the nasal airflow in nasal cavities with partially or totally resected middle turbinates. Based on those results, he recommended that the anterior inferior part of the middle turbinate should be reduced, and the posterior margin should be preserved to improve nasal airflow [18]. Further investigations in this field are certainly necessary.

Like all methods used for the study of nasal physiology, computational fluid dynamics had some limitations. First, it is very time-consuming simulation. After obtaining the image from CT scans, constructing the 3D model and performing the simulations required several days or weeks of work. In addition, there is the radiation exposure during CT scans. On the other hand, computed tomography scans are usually standard part of preoperative management. An appropriate alternative might be magnetic resonance imaging, but this approach is even more expensive and more time-consuming [19].

A significant challenge to computational fluid dynamics is the variable geometry of the nasal cavity, due to the periodic congestion of the nasal mucosa. These changes might determine the overall nasal resistance and modify the airflow in the nasal cavity [19]. But generally, all imaging methods and functional examinations of the nasal cavity (e.g., acoustic rhinometry or rhinomanometry) have these same limits. Our results (and previously discussed results of other authors) of different nasal airflows between the right and left nasal cavities in computational fluid dynamics model could be explained precisely by periodic congestion of the nasal mucosa, as computational fluid dynamics is not able to distinguish between dynamic and static cause of nasal obstruction and evaluate the development of nasal obstruction over time [14,15,16].

Therefore, if all the pros and cons of computational fluid dynamics are weighed, it could be indicated mainly in challenging cases (e.g., patients with significant discrepancy between the clinical findings and the patient’s subjective complaints), in which its results can potentially help (as a set of auxiliary indicators together with other methods) with clinical decision-making and with the right selection of the necessary extent of revision surgery considering different results of individual methods in one patient [20]. Another group of patients who could possibly benefit from computational fluid dynamics are patients who underwent septoplasty and/or turbinoplasty with no relief of their nasal obstruction [21]. In these cases, computational fluid dynamics results could reveal reasons for the lack of improvement in patients’ symptoms and determine other correct resection sites. In our particular case, partial resection of the middle turbinates or other intervention targeting the middle nasal meatus and the middle part of the common nasal meatus could be considered according to individual computational fluid dynamics results. These sites are not usually the aim during surgical therapy for nasal obstructions [21]. On the other hand, CFD alone may not correspond completely to the patient’s symptoms (like other examinations) and therefore may not accurately predict their disappearance after surgery.

A limit of our study is naturally the number of included patients, as all modeling was performed on one original CT scan of one patient. This issue did not allow us to draw broader conclusions from the modeling results that would apply to individual groups of patients or even to the entire population. We can only demonstrate its irreplaceable potential and benefit for this specific case to the diagnostic mosaic (together with the results of other diagnostic methods) in managing challenging cases of nasal obstruction. Gender, racial, and ethnic differences in nasal anatomy and function need to be considered as well [22]. Another limit of the study is that our CFD model does not take account of some parameters which play a role in respiratory dynamics, such as heat transfer from the air to the nasal mucosa, wall shear stress, airstream velocity, and streamlines [23].

Further investigations are certainly necessary to address all discussed issues. However, despite its limitations, computational fluid dynamics seems to have great potential for use in clinical practice, particularly in rhinosurgery, in carefully selected cases.

## 5. Conclusions

Nasal airflow was different between the right and left nasal cavities in our computational fluid dynamics model. The model showed the main routes of airflow in the inferior meatus and inferior part of the common meatus, but also surprisingly in the middle meatus and in the middle part of the common nasal meatus. These results indicate that the lower meatus and the lower part of common meatus should not be the only consideration in case of surgery for nasal obstruction in our patient. It documents that computational fluid dynamics surgical planning could enable individualized precise surgical treatment of nasal disorders. It could be mainly beneficial in challenging cases such as in patients with persistent nasal obstruction after nasal surgery, patients with empty nose syndrome, and in patients with discrepancies between the clinical findings and their subjective complaints.

## Figures and Tables

**Table 1:** The resistance to airflow in the different regions of nasal cavity (7 CT sections), expressed as the percentage difference in pressures between the individual sections (%).

Sections	Resistance (%)
1–2	95
2–3	49.5
3–4	19.5
4–5	2.31
5–6	5.04
6–7	0.43

**Table 2:** Evaluation of maximal and median airflow velocities in nasal passages, measured in 7 nasal regions identified on CT sections.

Section	Maximal Velocity v_max_ (m/s)	Median Velocity v_med_ (m/s)
1	11.8	3.09
2	8.34	2.09
3	4.64	1.21
4	3.70	0.69
5	2.99	0.79
6	1.71	0.62
7	1.14	0.83

## Data Availability

No data supporting reported results can be found.
